# Towards continuous non-invasive blood pressure measurements—interpretation of the vasculature response to cuff inflation

**DOI:** 10.3389/fphys.2023.1172688

**Published:** 2023-06-02

**Authors:** João Loureiro, Laura Bogatu, Lars Schmitt, Jorge Henriques, Paulo Carvalho, Gerrit J. Noordergraaf, Igor Paulussen, Jens Muehlsteff

**Affiliations:** ^1^ Centre for Informatics and Systems of the University of Coimbra, University of Coimbra, Department of Informatics Engineering, Coimbra, Portugal; ^2^ Department of Patient Care and Measurements, Philips Research, Eindhoven, Netherlands; ^3^ Department of Anesthesiology and Pain Management, Elisabeth-Tweesteden Hospital, Tilburg, Netherlands

**Keywords:** blood pressure, pulse arrival time, oscillometry, blood pressure surrogate calibration, hemodynamic model, pulse transit time, NIBP

## Abstract

Blood pressure (BP) surrogates, such as pulse transit time (PTT) or pulse arrival time (PAT), have been intensively explored with the goal of achieving cuffless, continuous, and accurate BP inference. In order to estimate BP, a one-point calibration strategy between PAT and BP is typically used. Recent research focuses on advanced calibration procedures exploiting the cuff inflation process to improve calibration robustness by active and controlled modulation of peripheral PAT, as measured via plethysmograph (PPG) and electrocardiogram (ECG) combination. Such methods require a detailed understanding of the mechanisms behind the vasculature’s response to cuff inflation; for this, a model has recently been developed to infer the PAT-BP calibration from measured cuff-induced vasculature changes. The model, while promising, is still preliminary and only partially validated; in-depth analysis and further developments are still needed. Therefore, this work aims to improve our understanding of the cuff-vasculature interaction in this model; we seek to define potential opportunities and to highlight which aspects may require further study. We compare model behaviors with clinical data samples based on a set of observable characteristics relevant for BP inference and calibration. It is found that the observed behaviors are qualitatively well represented with the current simulation model and complexity, with limitations regarding the prediction of the onset of the distal arm dynamics and behavior changes at high cuff pressures. Additionally, a sensitivity analysis of the model’s parameter space is conducted to show the factors that influence the characteristics of its observable outputs. It was revealed that easily controllable experimental variables, such as lateral cuff length and inflation rate, have a significant impact on cuff-induced vasculature changes. An interesting dependency between systemic BP and cuff-induced distal PTT change is also found, revealing opportunities for improved methods for BP surrogate calibration. However, validation via patient data shows that this relation does not hold for all patients, indicating required model improvements to be validated in follow up studies. These results provide promising directions to improve the calibration process featuring cuff inflation towards accurate and robust non-invasive blood pressure estimation.

## 1 Introduction

The current standard for clinical Non-Invasive Blood Pressure (NIBP) monitoring is based on sphygmomanometry utilizing a cuff at the upper arm. With the cuff, the transmural pressure across the brachial artery wall is changed, causing the volume of the brachial artery to oscillate with varying amplitude, which is detected as air pressure oscillations inside the cuff. The BP values are then inferred by empirical methods, namely, oscillometry. Depending on the patient’s state, this measurement is taken at intervals ranging from a few minutes to multiple hour intervals, implying the risk of not being able to continuously follow the hemodynamic state of a patient undergoing therapeutic intervention, particularly when it comes to not foreseeing (or entirely missing) critical events (hemorrhages, shock, among others), preventing timely and adequate therapy (Vincent et al., 2011).

For this reason, substantial research has been dedicated towards accurate and tendentially continuous NIBP measurement. In particular, the use of Blood Pressure (BP) surrogates, such as Pulse Wave Velocity (PWV), Pulse Transit Time (PTT), and Pulse Arrival Time (PAT), has been extensively explored (Sola et al., 2019). PAT is the time a pulse takes to travel from the heart to a peripheral artery, extracted as the time delay between the R-peak of the electrocardiogram (ECG) signal and a signal feature in a synchronously acquired photoplethysmogram (PPG) signal (e.g., from the finger), PTT is the time between two pulses measured at two artery locations measured with, for example, two PPG devices (Panula et al., 2023). While such measurements are widely available, PAT/PTT-based BP estimation methods are not being implemented in standard practice. A main limitation is the difficulty of estimating the BP surrogate calibration–e.g., an x ms change in PAT/PTT reveals a y mmHg change in BP. A large number of calibration strategies of various levels of complexity have been attempted, e.g., based on demographic data (Chen et al., 2009; Gesche et al., 2012), waveform feature analysis (Yoon et al., 2018), machine learning (Su et al., 2018), among others (Sola et al., 2019). However, there is still an unmet need for accurate and continuous NIBP measurement methods.

Recently, Bogatu et al. (2020) discussed the opportunities in estimating BP-surrogate calibration by utilizing the cuff in combination with other typically available signals such as the electrocardiogram (ECG) and photoplethysmogram (PPG). The cuff actively modulates the blood flow and pulse propagation along the artery distal to the cuff affecting, for instance, pulse arrival time (PAT) or pulse transit time (PTT). The PAT response to cuff inflation may, in principle, be representative of the PAT-BP relationship over a large BP range and, therefore, be used to establish a PAT-BP calibration with improved robustness, allowing for more accurate measurements. However, the correct interpretation of the measured cuff-induced change in PAT poses a challenge. Early models did not correctly explain the dynamics of the PAT response during cuff inflation, e.g., Yan and Zhang (2007) or Bresch et al. (2018). Recent clinical data provided an improved understanding of the true hemodynamic processes measuring the BP dynamics (Bogatu et al., 2021). The cuff decreases the transmural pressure at the brachial artery, causing an increase in PTT for this artery segment. At the same time, the mean arterial pressure (MAP) increases in the distal portion of the arm due to venous collapse, impacting the effective overall observed PAT behavior.

**FIGURE 1:**
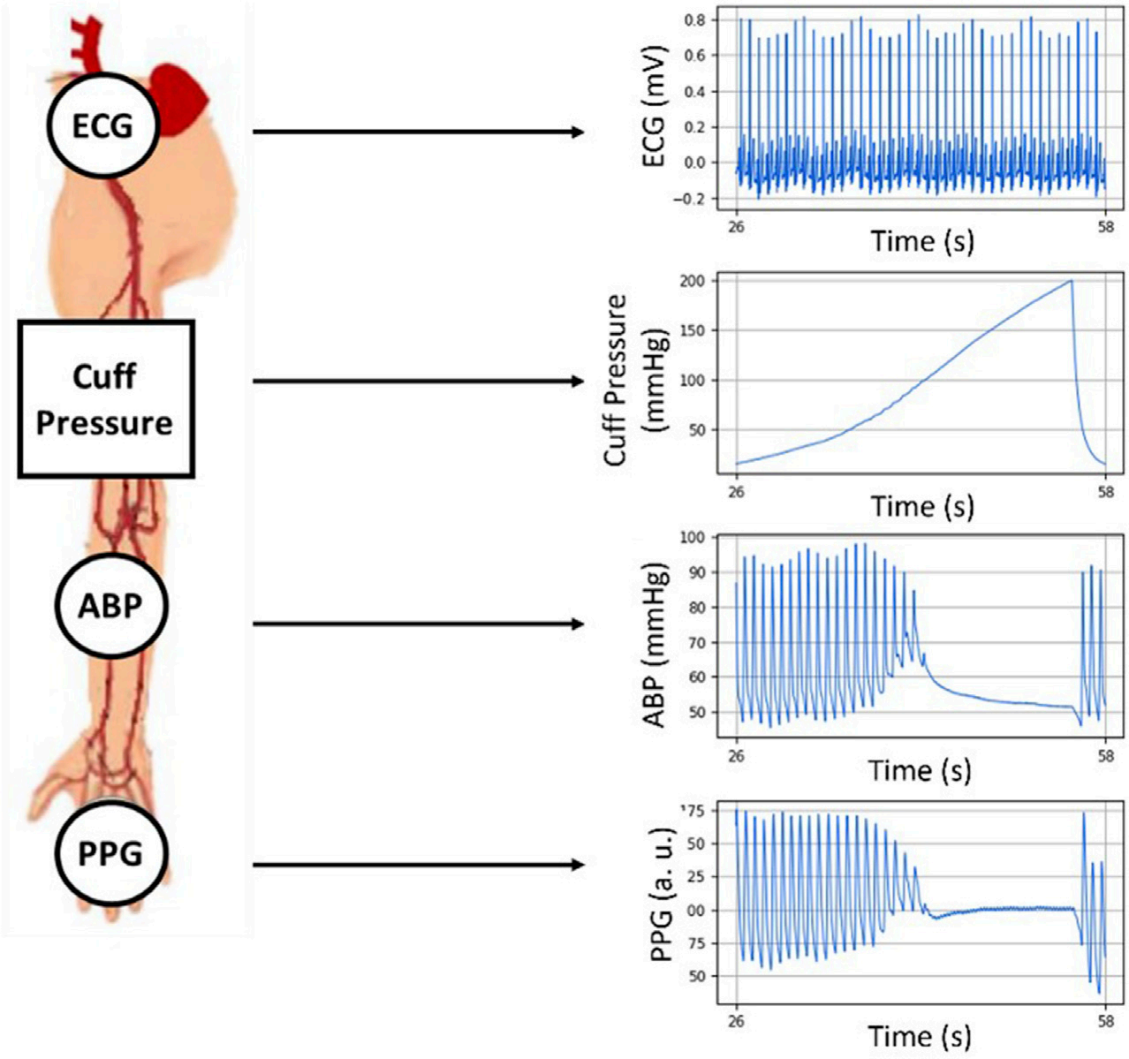
The setup utilized in this work acquiring ECG, PPG, the cuff air pressure and the invasively measured radial ABP. All signals are synchronously recorded during a cuff inflation.

The basic measurement configuration of this work is shown in Figure 1. It includes the simultaneous acquisition of an ECG signal, the pressure within an inflatable cuff at the upper arm, a finger-site PPG, and the invasive measurement of ABP in the radial artery providing an accurate measurement for comparison/validation.

This complex and dynamic PAT response to cuff inflation has been simulated via a hemodynamic model including the cuff and the distal arm by Bogatu et al. (2021), aggregating work from Drzewiecki et al. (1994), Bank et al. (1999), Seagar et al. (1984) and Babbs et al. (2012). This implementation incorporates the dynamic of the BP changes in the distal arm. Preliminary clinical evidence of an improved PAT-BP calibration performance for BP estimation has been reported.

The model, while promising, is still preliminary and only partially validated; in-depth analysis and further developments are still needed. This paper aims at a deeper understanding and critical evaluation of this simulation model in order to strategize possible improvements. Particular attention was given to the underlying dynamics during cuff inflation. For these purposes, we discuss the results of 1) a direct qualitative comparison between simulated and real-life distal BP and PAT data, as well as 2) a sensitivity analysis of the model, identifying and assessing the key variables that affect the distal arm’s vasculature response as observed in measurements. These will provide insights on the main factors to explore for future improvements in terms of robustness and performance of BP surrogate calibration.

The remainder of this work is structured as follows.

• In Section 2, the materials and methods employed for this research are described, starting from the simulation model under analysis, followed by the data collection and characterization strategy, as well as the sensitivity analysis that was performed.
• Section 3 encompasses the findings obtained over the course of the various analyses carried out in this work, including a brief description of the results along with their exposition via figure/table. We start by presenting the results of our qualitative comparison between simulated and real data, moving on to the outcomes of the sensitivity analysis.
• Our findings will be subject of discussion in Section 4, where a critical assessment of our work is conducted, highlighting the successes and limitations of our study, as well as their meaning regarding the current hemodynamic monitoring landscape.
• Lastly, Section 5 summarizes and concludes this work.

## 2 Materials and methods

### 2.1 Simulation model

**FIGURE 2:**
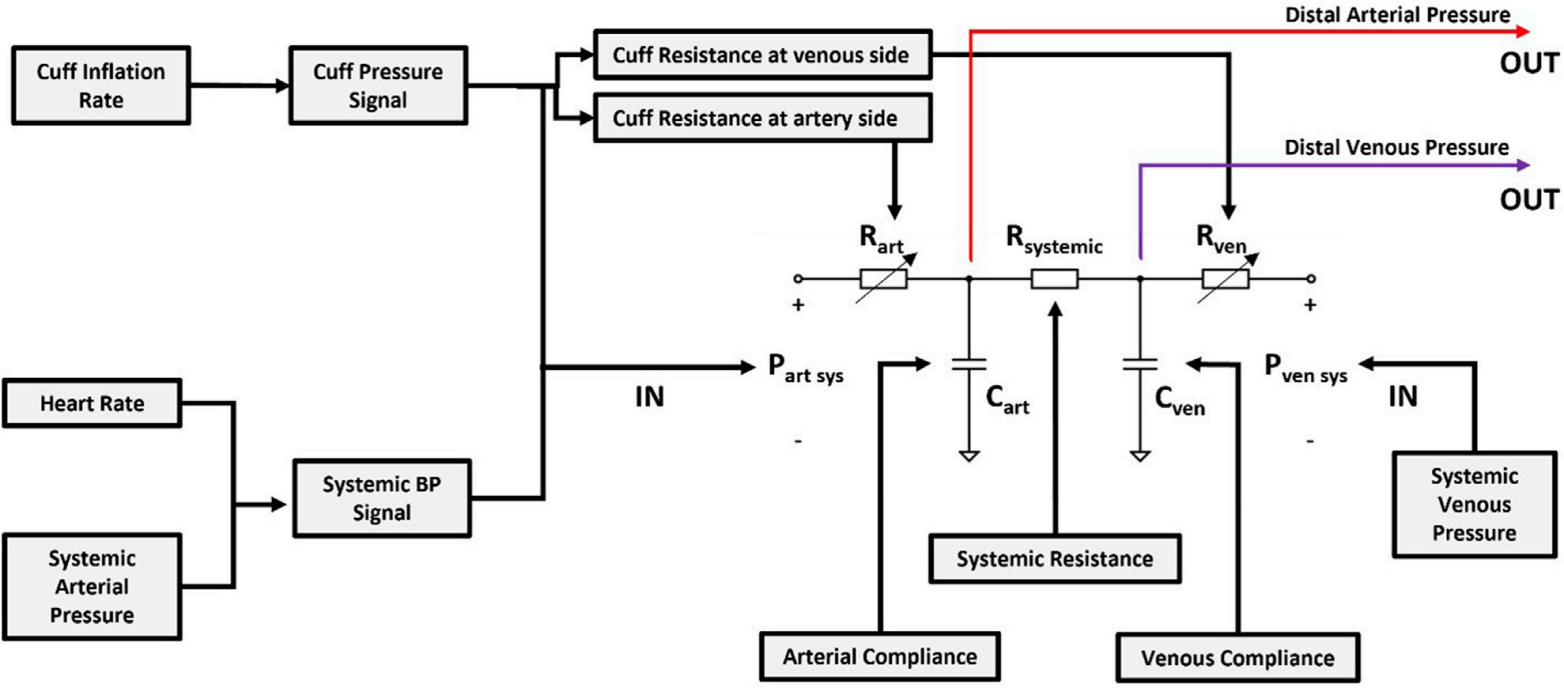
Overview of the distal arm circulation model.

An overview of the model analyzed in this work is shown in Figure 2 with input/output parameters and an internal resistor-capacitor (RC) network.

All model variables are listed in Table 1. The inputs of the model are systemic venous pressure, *P*_*ven sys*_, cuff inflation rate, and the systemic arterial pressure, *P*_*art sys*_, assumed as a sinusoidal signal defined by heart rate and systemic systolic and diastolic pressures. The arterial and venous pressures at the distal portion of the arm are the model’s outputs. The behavior of the distal BP signal is characterized by the systolic and diastolic pressure values measured at the superior and inferior peaks of the wave, respectively.

**TABLE 1:** List of variables and respective reference/control values for the distal arm circulation model.

Parameter	Units	References value
*a*	mmHg^-1^	0.03
*c*	mmHg^-1^	0.1
*d*	cm	0.08
Systolic Pressure	mmHg	120
Pulse Pressure	mmHg	40
Heart Rate	b.p.m	60
Cuff Inflation Rate	mmHg/s	6
Arm length	m	1
Cuff Length	m	0.14

The RC parameters have been modelled as follows: the resistance to blood flow across the portion of the brachial artery occluded by the cuff is represented by *R*_*art*_, estimated using the Poiseuille Formula as stated in (Eq. 1):

$$R_{art}\left(P_{tm}\right)=\frac{8\eta L_{cuff}}{\pi r{\left(P_{tm}\right)}^{4}}$$ (1)

where *η* is the blood’s viscosity, *L*_*cuff*_ is the cuff’s length, *P*_*tm*_ is transmural pressure, calculated as arterial BP (*P*_*art*_) minus cuff pressure (*P*_*cuff*_), and *r* (*P*_*tm*_) is the brachial artery’s radius as a function of transmural pressure across the arterial wall, which we may obtain from the arterial cross-sectional area given by (Eq. 2), introduced by Drzewiecki et al. (1994):

$$A\left(P_{tm}\right)=d\frac{\ln\left(aP_{tm}+3.3\right)}{1+e^{-cP_{tm}}}$$ (2)

with *a*, *c*, and *d* as model parameters. The venous resistance, *R*_*ven*_, has been found to be uncritical (Smink et al., 2019)—venous pressure typically ranges between 5 and 15 mmHg (Raju, 2019), being fixed at 10 mmHg for this framework. We can therefore assume that the vein is fully collapsed at a cuff pressure of around 30 mmHg (*R*_*ven*_ virtually infinite). For the systemic resistance of the arm, *R*_*systemic*_, Alastruey et al. (2006) reported values of about 100 mmHg·s/mL. Arm length is fixed at 1 m.

Two capacitances/compliances were included in the model: *C*_*art*_ and *C*_*ven*_, arterial and venous compliance, respectively. *C*_*art*_ is defined with a fixed value of 0.03 mL·mmHg-1 (Alastruey et al., 2006) and *C*_*ven*_ is defined as approximately 30 times larger than *C*_*art*_ (Gelman et al., 2008).

The model illustrated in Figure 2 can be represented via the following equations:

$$\frac{dP_{art\;distal}}{dt}=\left(\frac{P_{art\;sys}}{R_{art}}-P_{art\;distal}\left(\frac{1}{R_{art}}+\frac{1}{R_{systemic}}\right)+\frac{P_{ven\;distal}}{R_{systemic}}\right)\frac{1}{C_{art}}$$ (3)

$$\frac{dP_{ven\;distal}}{dt}=\left(\frac{P_{art\;distal}}{R_{systemic}}-P_{ven\;distal}\left(\frac{1}{R_{systemic}}+\frac{1}{R_{ven}}\right)+\frac{P_{ven\;sys}}{R_{ven}}\right)\frac{1}{C_{ven}}$$ (4)

The simulation of the interaction between the cuff inflation and pulse propagation at the cuff site is complemented by the modulation of the hemodynamic behavior in the distal portion of the arm. A good agreement between the observations obtained from a limited sample of patient data and model simulations was reported by Bogatu et al. (2021).

### 2.2 Clinical data acquisition and comparison with model output

Clinical data was collected from four anesthetized and mechanically ventilated patients (ages: 45, 58, 66, and 70 years old), using a sensor arrangement as shown in Figure 1. It consists of an ECG, a brachial blood pressure cuff at the upper arm, a radial intra-arterial line (ABP), and a finger PPG. The patients underwent invasive non-cardiac surgery at the time of the recordings. The data collection process was approved by the MEC-U ethical committee for this study (St. Antonius Ziekenhuis, Koekoekslaan 1, 3430 EM Nieuwegein, NL. Approval W19.046), and it was carried out at the Elisabeth Tweesteden Ziekenhuis hospital in Tilburg, NL. Each patient gave written informed consent. The signals were recorded using a Philips MP50 patient monitor and custom data logging software. The ECG signals were recorded with a sampling frequency of 500 Hz whereas the cuff pressure, ABP, and PPG signals were recorded with a sampling frequency of 125 Hz. All the signals were simultaneously recorded.

The variations in PAT and PTT caused by the inflation of the cuff are calculated over two vascular segments.

• Heart to finger site: *ΔPAT*_*ECG–PPG*_ (*P*_*cuff*_) is calculated as the change in delay between the R-peak of the ECG signal and the foot of the PPG waveform as *P*_*cuff*_ increases.
• Radial to finger site: *ΔPTT*_*ABP–PPG*_ (*P*_*cuff*_) is calculated as the change in delay between the foot of the ABP waveform and the foot of the PPG waveform as *P*_*cuff*_ increases.

The difficult assessment of the heart’s pre-ejection period (PEP) has been characterized as a drawback of the direct application of PAT measurements in BP estimation (Pilz et al., 2023). The presented calibration method eliminates this factor by interpreting the PAT/PTT variations, given that they are generated due to the action of the cuff and PEP remains constant throughout the inflation.

**FIGURE 3:**
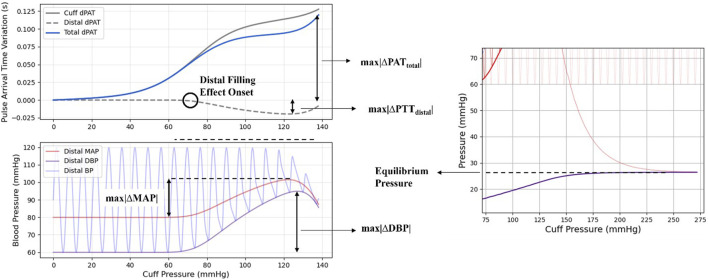
Illustration of the metrics as used for this study.

The dynamics taking place in the portion of the arm distal from the cuff are the focus of the model under study. The early venous collapse at the cuff site causes an increase of BP in the distal arm due to continued blood flow at intermediate cuff pressures, which can be measured via invasive methods and is reflected in deviations in the PAT/PTT measurements. With the purpose of describing this distal filling effect, a series of metrics were defined and are explained below and illustrated in Figure 3.

• The maximum drop in distal PTT variations (ΔPTT) caused by the distal filling effect - ***max|∆PTT***_***distal***_ ***|*** - measured via the time interval between the pulse detected at the ABP site and the pulse detected at the finger site through the valleys of the ABP and PPG waveforms.
• The maximum total PAT change (ΔPAT) - ***max|∆PAT***_***total***_ ***|*** - measured via the time interval between the R-peak of the ECG signal and the pulse detected at the finger site at the valley of the PPG waveform.
• The maximum change in distal MAP - ***max|∆MAP|*** - measured as the difference between the maximum measured MAP value during a cuff inflation and the MAP value at the beginning of the cuff inflation.
• The equilibrium pressure - ***P***_***eq***_–measured as the value to which blood pressure in the distal arm asymptotically tends at cuff pressures that cause arterial and venous occlusion in the upper arm.
• The maximum change in distal Diastolic Pressure (DBP) - ***max|∆DBP|*** - measured as the difference between the maximum measured DBP value during a cuff inflation and the DBP value at the beginning of the cuff inflation.
• The difference between MAP and cuff pressure at the onset of the distal filling effect - ***∆PTT***_***onset***_–calculated as the difference between systemic MAP and the cuff pressure at the onset of the observed decrease in ΔPTT.

These features have been also investigated in the sensitivity analysis, as discussed in the next section.

### 2.3 Sensitivity analysis

The sensitivity analysis carried out in this work is based on the variance-based method for Global Sensitivity Analysis (Sobol, 2001; Saltelli et al., 2004), as it is model-independent and allows us to analyze non-linear and non-monotonic functions and models. This method provides a structured approach to identify the most influential parameters of a simulation model via their contribution to the overall model output variance. The technique is based on the decomposition of the model variance into Sobol Indices. These Indices reflect the multiple order contributions to output variance from the various parameter subsets, beginning with the first order Sobol Indices that correspond to the contribution from each individual parameter, moving to the second-order Sobol Indices that reflect interactions between pairs of parameters, and so on. The total-order Sobol indices reflect the overall contribution each parameter has to the variance of the output. Assuming a black-box model with independent input variables represented by an integrable function:

$$Y=f\left(X\right)=f\left(X_{1},...,X_{n}\right)$$ (5)

where Y is the model output (or objective function) and $\left(X_{1},...,X_{n}\right)$ is the input variable set. We can decompose the function *f* into summands of increasing dimensionality:

$$Y=f\left(X_{1},...,X_{n}\right)=f_{0}+\sum_{i}^{n}f_{i}\left(X_{i}\right)+\sum_{i<j}^{n}f_{ij}\left(X_{i},X_{j}\right)+\cdots +f_{1,...,n}\left(X_{1},...,X_{n}\right)$$ (6)

and, subsequently, decompose the variance of Y into the summand of the variances of individual parameters and their interactions (Sobol, 1993), as in Eq. 5:

$$V\left(Y\right)=\sum_{i}^{n}V_{i}+\sum_{i<j}^{n}V_{ij}+\cdots +V_{i,...,n}$$ (7)

We can then calculate the Sobol indices for a parameter *X*_*i*_ as the ratio of the partial variance which the parameter contributes to the total variance *V*:

$$First\;Order\;Sobol\;Index:S_{i}=\frac{V_{i}}{V}$$ (8)

$$Second\;Order\;Sobol\;Index:S_{ij}=\frac{V_{ij}}{V}$$ (9)

$$Total\;Order\;Sobol\;Index:S_{Ti}=S_{i}+\underset{j\neq i}{\sum }S_{ij}+\cdots$$ (10)

Based on these indices, we draw conclusions on the influence of a parameter on the model. On relative terms.

• If the first order index is high (close to 1), the parameter has a strong influence on the model by itself.
• If the total order index is low (close to 0), the parameter has a weak impact on the model.
• If the first order index is low and the total order index is high, the influence that the parameter has on the model is impacted by interactions with other parameters.

Naturally, the method described above is analytically feasible for simple, analytically trackable models, which is not the case for most. However, for complex and highly non-linear models such as the one at study, the individual variances in Eq. 7 can be estimated via Monte Carlo (or quasi-Monte Carlo) sampling, solely relying on the output of the model, as demonstrated by Sobol (1993).

The list of parameters analyzed in the course of the sensitivity analysis is shown in Table 2 including their respective assumed distributions. There is limited prior information on the arterial collapse parameters *a* and *c*, which have been assigned based on clinical data as found by Bogatu et al. (2021). Regarding the Systolic Pressure (SBP) and Pulse Pressure (PP) distributions, and given that this method assumes independent input parameters, we used Gaussian distributions. For both variables, the means and standard deviations produce realistic samples, in line with the European Society of Hypertension - European Society of Cardiology (ESC) Guidelines for the Management of Arterial Hypertension (Williams et al., 2018). These distributions also provide samples characteristic of hypotensive and hypertensive patients. The sensitivity analysis also evaluates the robustness of these assumptions. Cuff length and inflation rate were set as uniformly distributed given conditions from practice. It must be noted that these settings are treated as variables in this study to assess their value in further experimental protocols, as they were kept constant in the course of the clinical data collection. Remaining constraints and parameters, in particular the internal RC components of the model, were fixed as described in the previous section.

**TABLE 2:** Assumed parameter constraints; Normal Distribution: N (µ, σ); Uniform Distribution: U (lower limit, upper limit).

Parameters (Units—Type)	Distribution
*a* (mmHg-1)	U (0.017, 0.035)
*c* (mmHg-1)	U (0.08, 0.14)
Systolic Pressure (mmHg)	N (125, 15)
Pulse Pressure (mmHg)	N (40, 5)
Cuff Inflation Rate (mmHg/s)	U (4, 8)
Cuff Length (m)	U (0.1, 0.18)

The parameter sets are generated according to a quasi-random, low-discrepancy sampling method (Saltelli, 2002). For each set, the output of the model is computed and its characteristics are registered. The metrics for this quantitative assessment are the same as discussed in Section 2.3 and illustrated in Figure 3.

The implementation of the methods required for this analysis is based on the SALib–Sensitivity Analysis Library in Python (Herman et al., 2017; Iwanaga et al., 2022). The outputs of the model with each parameter set were stored for use in the analysis itself and our own quantitative assessment.

## 3 Results

### 3.1 Measured signal vs. model output signal comparison

#### 3.1.1 Distal BP

**FIGURE 4:**
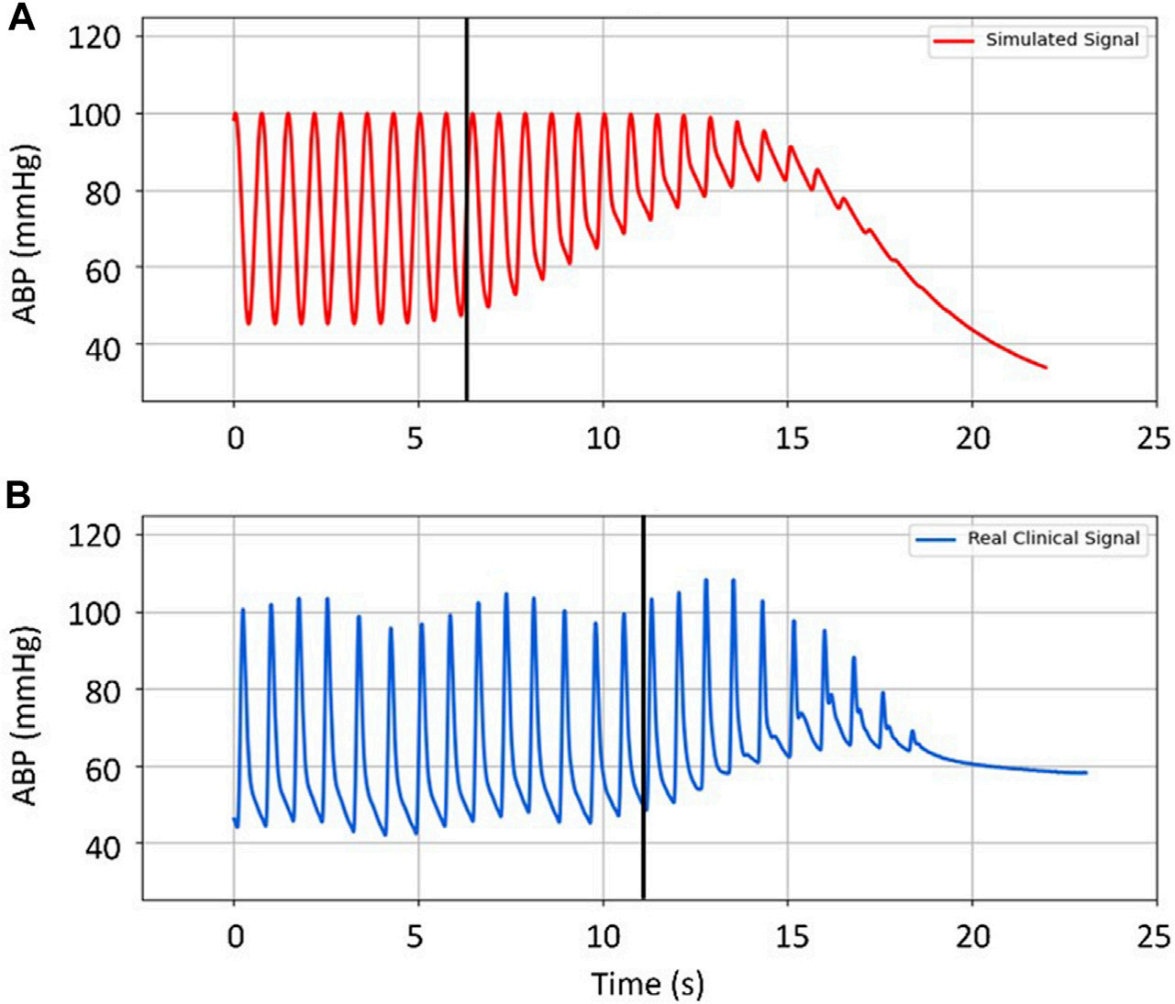
Comparison between **(A)** simulated distal arterial BP, and **(B)** real clinical data from a patient. Black vertical lines mark the onset of the filling effect (the moment when distal pressure starts to differ from systemic pressure). Input parameters for the simulated data are the same as the actual patient’s: HR: 1.4 Hz (∼84 b.p.m.); BP: 100/45 mmHg; Cuff Inflation Rate: 6 mmHg/s; Arterial Collapse Parameters (*a*, *c*, and *d*): reference values, see Table 1.

An example of the qualitative comparison between clinical signal samples and simulated data assuming similar physiological conditions, namely, BP and heart rate, is shown below with a cuff inflation at t = 0 s. The goal is to identify differences in ABP signal behaviors. This comparison is shown in Figure 4.

Visually, the dynamics of BP, the increases in DBP and MAP as well as the decrease in the SBP at the radial location, are well reproduced by the model. However, it is noticeable that the increase in DBP and MAP begins sooner in the simulation than in the clinical data, also reaching higher values (∼40 vs. ∼25 mmHg, respectively). By contrast, the decrease in SBP seems to be smaller in the model than in the real case. Different dynamics regarding the tendency towards equilibrium pressure are evident between simulation and clinical data. It should be noted that, by design, the model does not take into account difficult to measure or to control parameters such as cardiac output and peripheral resistance.

#### 3.1.2 PAT/PTT

**FIGURE 5:**
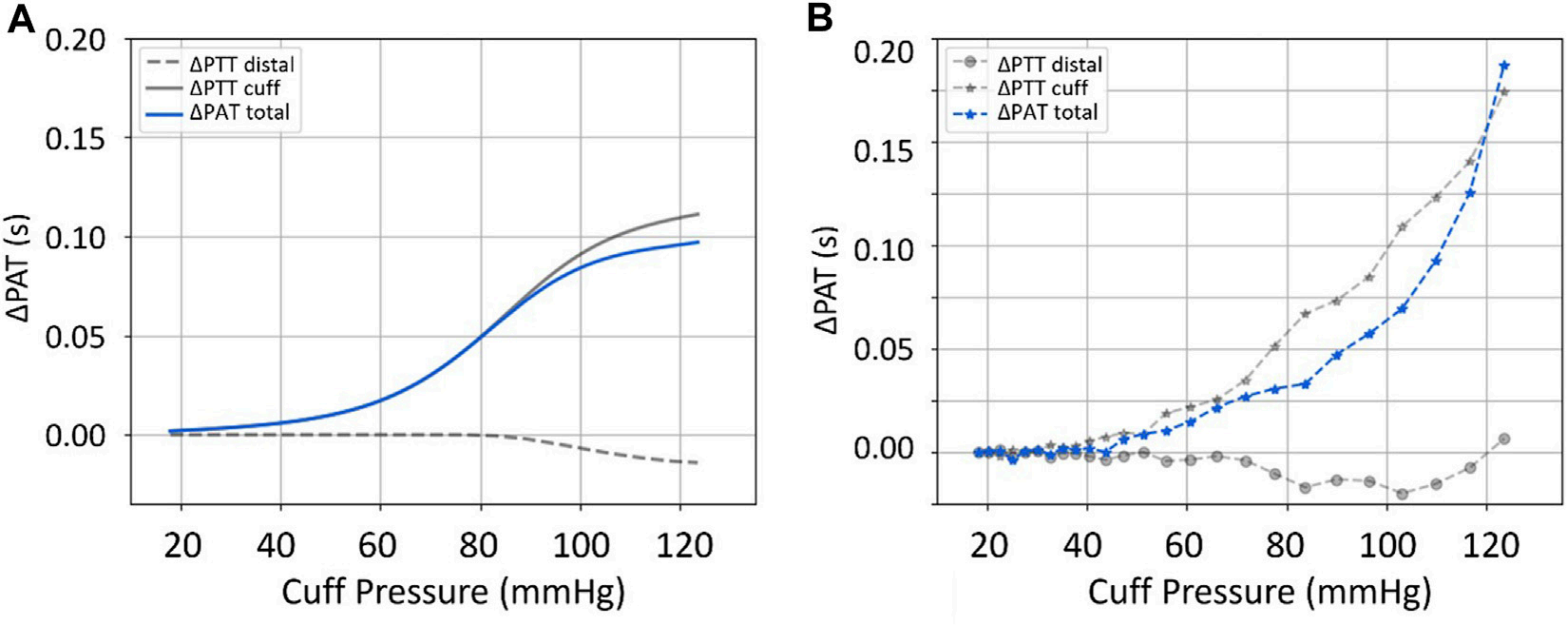
Comparison between **(A)** simulated, and **(B)** real measurements of *ΔPTT*_*cuff*_, *ΔPTT*_*distal*_, and *ΔPAT*_*total*_. Input parameters for the simulated data are an approximation of the actual patient’s: HR: 1.22 Hz (∼73 b.p.m.); BP: 123/75 mmHg; Cuff Inflation Rate: 6.8 mmHg/s; Arterial Collapse Parameters (*a*, *c*, and *d*): reference values, see Table 1.

In addition to the distal ABP signals, ΔPAT and ΔPTT behavior is also investigated. The measured ΔPAT/PTT signals obtained from the ECG, ABP and PPG recordings, as well as the simulated ΔPAT/PTT signals are computed as described by Bogatu et al. (2021). Figure 5 provides a side-by-side comparison between an example of a clinical measurement (right diagram) and the equivalent simulation results (left diagram), following the same strategy as before. In this case, the total resulting measurements (*ΔPAT*_*total*_) can be decomposed in two components: *ΔPTT*_*cuff*_, corresponding to the change in PTT cause only by action of the cuff inflation, and *ΔPTT*_*distal*_ which is cause by the increase in DBP and MAP due to the filling effect. Key observations include: 1) the distal filling effect starts at lower cuff pressure (∼60 mmHg) with higher values of ΔPAT reached in the clinical data compared to the simulation; 2) there is an increase of distal ΔPTT at the end of the inflation, not covered by the simulation; 3) the ΔPAT measurements tend to higher values in the clinical data, compared to the model.

**FIGURE 6:**
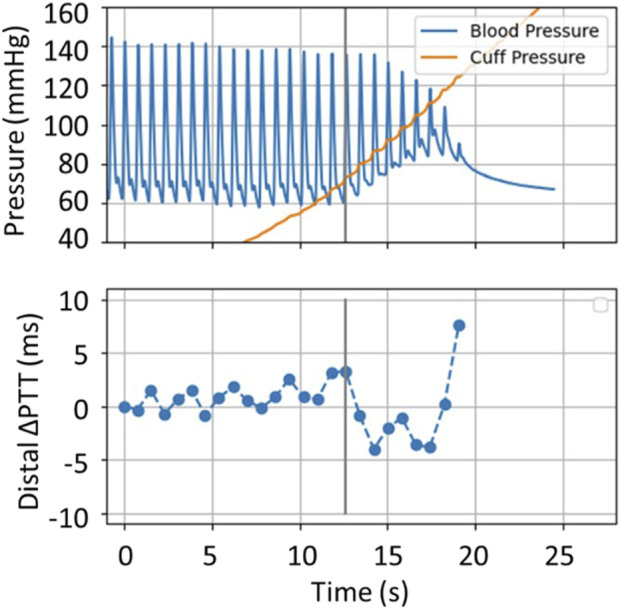
Simultaneous recordings of distal BP signal and distal ΔPTT from the clinical dataset. The vertical line marks the onset of the distal filling effect.

In Figure 6 it is observed that the onset of the MAP increase and the onset of ΔPTT occur simultaneously in the clinical data. This simultaneous onset is observed across the database, confirming that the model represents this process well (in the simulations, onset of MAP and onset of *ΔPTT*_*distal*_ are modelled to occur simultaneously). However, the model cannot be used in the current state to predict when the onset will occur along the cuff inflation. As shown in the example in Figure 4, the onset of the distal effect happens at a different moment in the simulation compared to measured data, whereas in Figure 5 the onset happens later in the simulation. The determinants of this process are explored in the sensitivity analysis.

Overall, the behaviors are qualitatively well represented via the model; the model is an acceptable representation of the core hemodynamic processes happening as result of the cuff inflation. However, significant differences can be observed in amplitudes and timing of signal changes, implying the existence of fundamental inaccuracies/limitations.

### 3.2 Sobol sensitivity analysis

The sensitivity analysis outputs the Sobol/Sensitivity Indices that reflect the influence of each parameter on the evaluated outcomes. The resulting first and total order Sobol Indices, respectively marked as S1 and ST, are listed below, in Table 3.

**TABLE 3:** First (S1) and Total (ST) Order Indices from the sensitivity analysis.

Parameter	*max|∆PTTdistal|*		*max|∆PATtotal|*		*max|∆MAP|*		*Peq*		*max|∆DBP|*		*∆PTTonset*	
	S1	ST	S1	ST	S1	ST	S1	ST	S1	ST	S1	ST
*a*	0.0283	0.0352	0.0243	0.0512	0	0	0.0008	0.0009	0	0.0003	0.0309	0.0317
*c*	0.0023	0.0038	0.0435	0.0881	0.0075	0.0079	0.0079	0.0082	0.0017	0.0021	0.0483	0.0540
Systolic Pressure	0.3519	0.3678	0.0331	0.0599	0.1495	0.1506	0.7729	0.7879	0.1588	0.1612	0.1490	0.1604
Pulse Pressure	0.5857	0.5974	0.0005	0.0138	0.8150	0.8161	0.0047	0.0047	0.8179	0.8190	0.7277	0.7468
Cuff Inflation Rate	0.0153	0.0160	0.0960	0.1330	0.0262	0.0275	0.1988	0.2145	0.0193	0.0214	0.0220	0.0313
Cuff Length	0	0	0.7577	0.7616	0	0	0	0	0	0	0	0

The results show that, concerning the model, SBP and PP are responsible for the majority of the variance of the outputs. SBP is the dominant parameter in relation to the equilibrium pressure *P*_*eq*,_ individually accounting for over 77% of its variability, and PP is the most influential factor for the observed decrease in distal ΔPTT, and the onset of the distal effect (*∆PTT*_*onset*_), as well as for increases in DBP and MAP, being individually responsible for ∼57%, ∼73%, ∼82%, and ∼82% of their variability, respectively. The magnitude of modelled ΔPAT (represented by *max|∆PAT*_*total*_ *|*) is mainly driven by cuff length, being more than 75% dependent on it, as compared to other parameters. The results show clearly that parameter *a* is the least influential parameter of the investigated set, never accounting for more than 3.09% for the variability whereas parameter *c* has some influence on *max|∆PAT*_*total*_ *|* and *∆PTT*_*onset*_. *P*_*eq*_ is found to also be ∼20% dependent on the cuff inflation rate. Finally, the differences between the First and Total Order indices are, albeit existent, not significative when compared to their overall magnitude, suggesting that higher order indices are not relevant for analysis.

**FIGURE 7:**
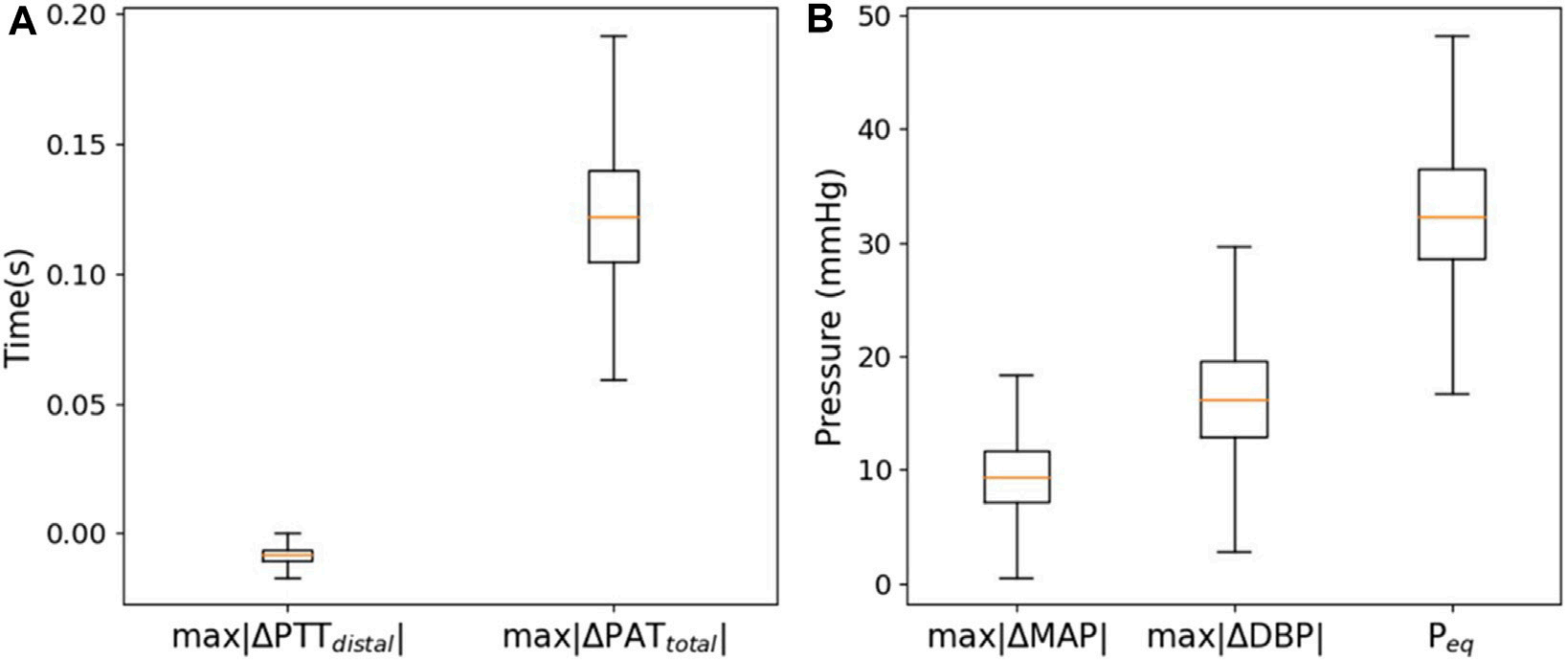
Boxplots of the generated **(A)** *max|∆PTT*_*distal*_ *|* and *max|∆PAT*_*total*_ *|*, and **(B)** *max|∆MAP|*, *max|∆DBP|*, and *P*_*eq*_.

Figure 7 illustrates model output characteristics for all simulated parameter ranges. The figure reveals that signals generated via the model fall within realistic physiological ranges; also indicating that model parameter ranges (Table 2) are representative of realistic values.

#### 3.2.1 Effects of cuff length and inflation rate in the simulated response

**FIGURE 8:**
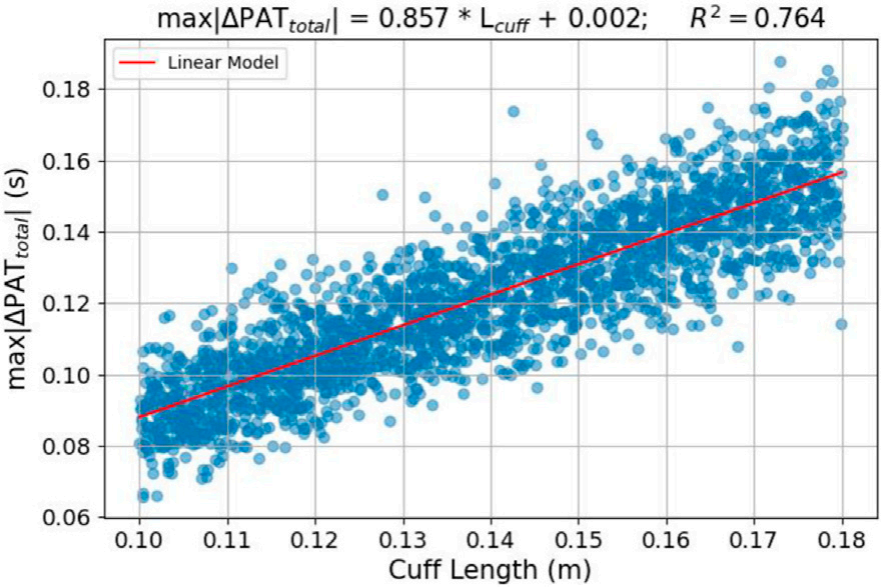
Relation between cuff length and *max|∆PAT*_*total*_ *|* and cuff length–scatter plot and linear model fit of the sampled simulation sets.

The results of the sensitivity analysis and the sampled parameter sets allow the analysis of hypotheses regarding dependencies between characteristics of the model outputs and its input variables. First, the dependencies between the experimentally controllable cuff length and inflation rate and the PAT/PTT response of the model are assessed. It is found that cuff length strongly determines the measured ΔPAT and *max|∆PAT*_*total*_ *|*. Figure 8 illustrates *max|∆PAT*_*total*_ *|* as simulated via the model plotted with respect to corresponding cuff length for all parameter sets included in the analysis. A strong linear dependency is found with an *R* ^2^ = 0.76.

**FIGURE 9:**
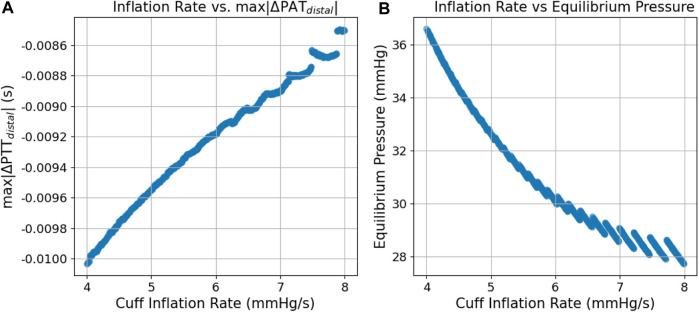
Scatter plots of the simulated relation between cuff inflation rate and **(A)** *max|∆PAT*_*total*_ *|* and **(B)** *P*_*eq*_.

Figure 9 illustrates the expected changes in distal vasculature response to different cuff inflation rates; *P*_*eq*_ and *max|∆PTT*_*distal*_ *|* are plotted with respect to cuff inflation rate. It is clear that slower inflations induce a more pronounced distal effect in *P*_*eq*_. In terms of *max|∆PTT*_*distal*_ *|*, the inflation rate can alter the *max|∆PTT*_*distal*_ *|* response by 1.5 millisecond; it is a small effect and it might be a challenge to measure.

#### 3.2.2 BP value vs. distal response correlation

**FIGURE 10:**
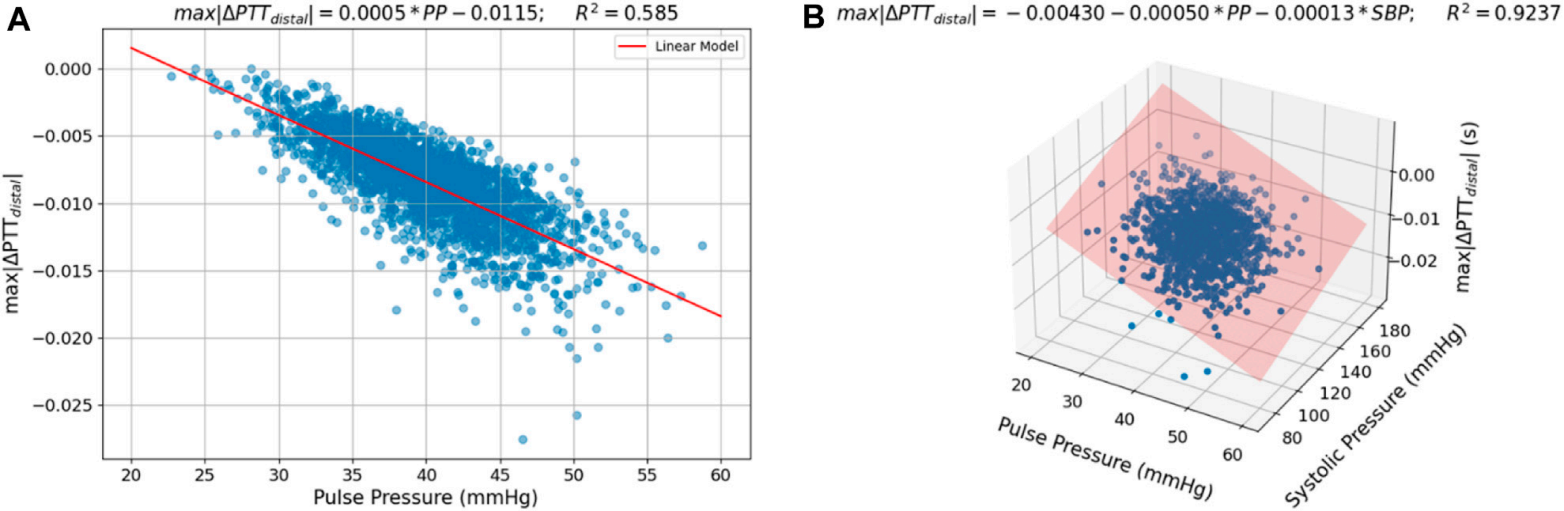
Scatter plots of the relation between **(A)** PP and *max|∆PTT*_*distal*_ *|* and **(B)** PP and SBP and *max|∆PTT*_*distal*_ *|* in the sampled parameter sets with a fitted linear model.

The sensitivity analysis (Table 3) reveals that the vasculature response to cuff inflation is considerably influenced by BP; namely, SBP and PP. We therefore investigate a measurement strategy based on the link between the BP value and the expected change in distal PTT, characterized by *max|∆PTT*_*distal*_ *|*. Figure 10 illustrates this connection - *max|∆PTT*_*distal*_ *|* is plotted against the corresponding PP value in a) and against the respective PP and SBP values in b). It is observed that a linear model can be used to characterize this link with good accuracy, particularly in b), as an *R* ^2^ of 0.9237 is determined.

The PP value could therefore be used as an index for the expected change in distal ΔPTT during cuff inflation, in this way allowing for improved estimation of brachial ΔPTT as measured non-invasively via ECG-PPG combination.

**FIGURE 11:**
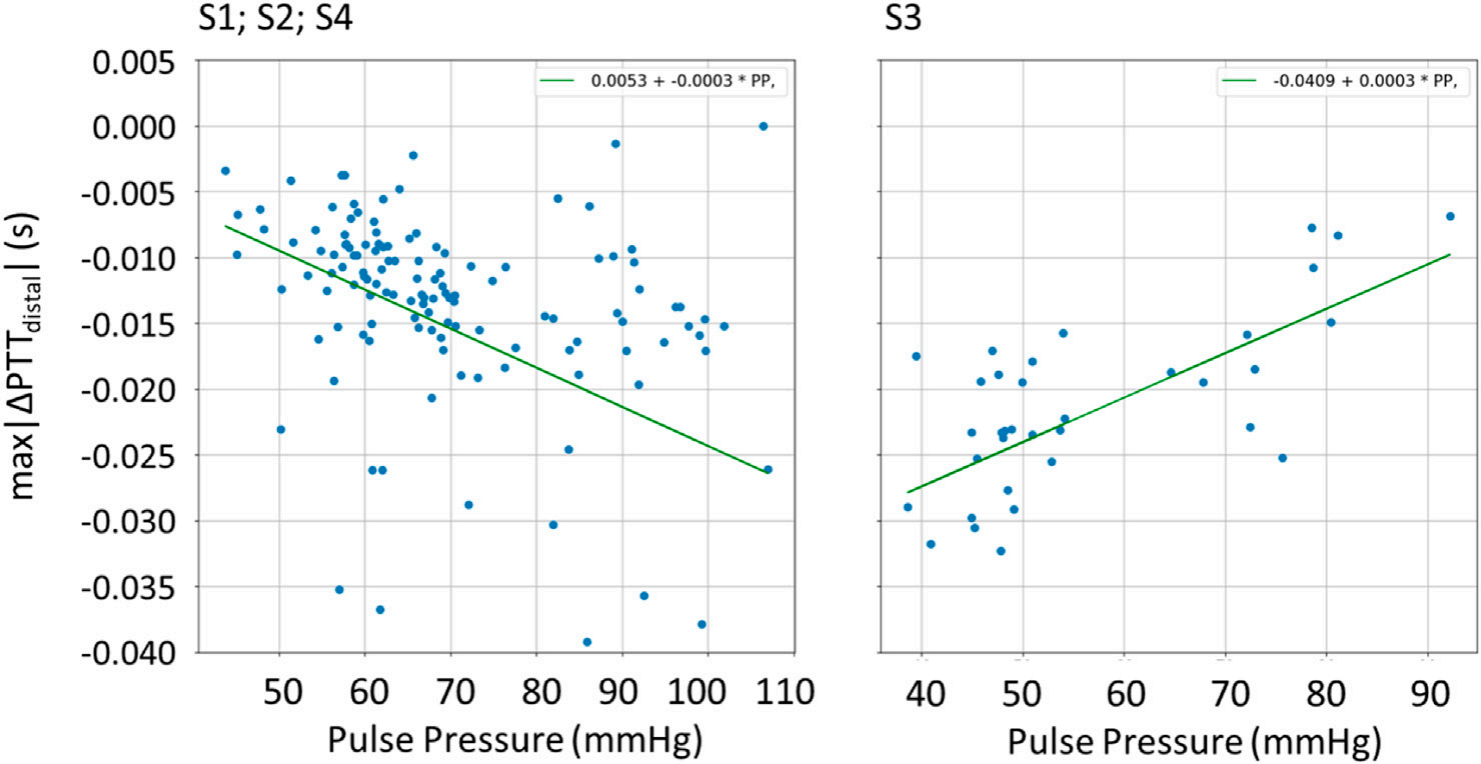
Scatter plot of the relation between PP and *max|∆PTT*_*distal*_ *|* for the patients S1, S2, S3, and S4, with fitted linear function.

This relationship was also explored in the patient data. The results are present in Figure 11, showing the dependency between *max|∆PTT*_*distal*_ *|* and PP as obtained from the recorded signals for the 4 evaluated subjects, S1, S2, S3, and S4. As shown, for S1, S2, and S4, lower PP is associated with lower magnitude measurements of *max|∆PTT*_*distal*_ *|*. This relationship, however, is not verified for S3, where the opposite seems to happen.

## 4 Discussion

### 4.1 Comparison between clinical data and model outputs

#### 4.1.1 Distal BP

Clinical data is compared by visual inspection with simulated data generated with comparable BP and heart rate values and cuff inflation rate and length. We find that the model is an acceptable representation of the core hemodynamic processes happening in the portion of the arm distal to the cuff, which includes the increases in DBP and MAP and the decrease in SBP as observed in the clinical data. From a physiological point-of-view, the observed dynamics are sound. A number of measurement stages can be identified: 1) Cuff pressure value is below systemic venous pressure–no changes occur; 2) Cuff pressure increases beyond systemic venous pressure–vein collapses, flow out of the limb is stopped, buildup of blood begins to occur in the limb from the artery; 3) Cuff pressure approaches systemic systolic pressure–minimal amount of blood flows into the limb at each heart-beat, a decrease in the distal systolic pressure is observed; 4) Eventually, blood flow is stopped–arterial and venous pressures tend towards an equilibrium value. The arterial pressure decreases via an exponential decay function.

However, differences can be observed in amplitudes and timing of signal changes. For example, shifts in DBP and MAP happen earlier and with different magnitude in the model than in the patient data. Such discrepancies may arise from two reasons: 1) the model in this state is not sufficiently complex to represent the full dynamics, such as the venous return, and the outcomes will differ from the patient measurements regardless of the chosen parameters for the simulation; 2) model constraints or incorrect assumptions on parameter ranges, particularly when it comes to the arterial collapse parameters and the distal arm vascular factors (resistances and compliances), both of which were obtained from very limited patient data, may explain differences in morphology of the signals and could be corrected via additional clinical measurements.

#### 4.1.2 PAT/PTT

Observation of ∆PAT and ∆PTT data from the simulation framework and the clinical dataset reveals differences concerning the response to cuff-induced variations in transmural pressure. In the example from Figure 5, we see similar qualitative behaviors observed in the simulation and clinical data, albeit with different timings and magnitudes. However, the model is not able to reproduce the clinical data even qualitatively at high cuff pressures, indicating either the need for model improvements and/or possibly inaccurate assumptions regarding constraints and parameter ranges. The PAT-BP calibration depends significantly on the correct understanding of these phenomena, towards accurate and personalized calibration procedure for cuffless and continuous BP measurement.

Upon observation of the complete set of clinical data samples, we find that the onset of the change in BP and the onset of the change in distal ∆PTT happens simultaneously, which is well depicted in the current model. Still, model improvements are needed to better characterize when the onset occurs with respect to cuff pressure. Finally, based on observations from the clinical data, these measurements require improved robustness given their low signal-to-noise ratio (SNR).

### 4.2 Sensitivity analysis

The results of the sensitivity analysis outline the major influence of SBP and PP. In contrast, the arterial collapse parameter *a* has only a relatively minute effect on the model’s behavior. As a consequence, this parameter could probably be set to a constant value when using the Bayesian technique for BP inference as it was introduced by Bogatu et al. (2021). Parameter *c*, despite also not being much influent from a global perspective, still has a noticeable impact under constant cuff length and inflation rate, meaning it should be included in the current estimation framework.

Another relevant finding deals with the impact of cuff length and inflation rate, which both can be easily changed in practice, suggesting a point of optimization of the measurement procedure, discussed more in depth in the following section.

An important result is the global impact of the BP parameters (SBP and PP) across most characteristics of the vasculature response to cuff perturbation.

Overall, we find that the characteristics of the outputs of the simulations are all well determined by one to three parameters each, indicating possibilities for simplified modelling, yet to be verified with clinical data. These results also indicate which parameters we must keep in mind when optimizing particular aspects of the model. It should also be noted that despite the high non-linearity of the simulation, higher order interactions between the parameters are not significant which could be an implication of the relatively low complexity of the framework.

As shown in Figure 7 via boxplots, the results indicate that outputs of the model reflect a realistic behavior over the entire parameter space. This further establishes the model as a useful tool for the representation of the complex hemodynamic processes at play in a global sense, albeit with room for improvement as has been discussed in this work.

#### 4.2.1 Effects of cuff length and inflation rate in the simulated response

The results from the analysis performed on the outputs from the sampled parameter sets reveal dependencies between the cuff length and inflation rate on the behavior of the model. Longer cuff lengths provide larger PAT changes in the course of the cuff inflation. This behavior can be interpreted physiologically–a longer cuff length covers a larger arterial segment. Along this arterial segment the artery is off-loaded and therefore the effective distally measured pulse wave velocity (PWV) is reduced (resulting in an increase in PAT). This result has practical implications on the PAT-BP calibration framework. A longer cuff could be used to increase the changes in PAT caused by the cuff inflation, improving the measurement’s SNR.

Clear effects caused by the inflation rate are also visible–on faster inflations, the drop in ∆PTT that characterizes the distal filling effect is smaller, and the theoretical equilibrium pressure is also lower. It should be noted that the scale of these differences (∼1 millisecond), despite small, is still significant within the scope of the distal ∆PTT measurements. This behavior is physiologically sound, as a slower inflation translates into more blood pumped in the limb (outflow of blood is stopped via brachial vein collapse, while inflow of blood via artery continues). In practice, slower inflation rates provide more PAT measurement points, as more heartbeats are included in the inflation time window, meaning that a greater amount of data is available for the Bayesian inference of the *a* and *c* parameters, potentially improving its accuracy. However, slower inflation rates also result in the amplification of the distal filling effect, adding uncertainty in a scenario where only non-invasive data sources would be available, which could have possible implications even in standard practice oscillometry. Further work is needed to find the optimal inflation rate for each case, balancing the amount of collected data with the intensity of these effects.

In addition, due to the impact this parameter has on the equilibrium pressure, being responsible for roughly 20% of its variability, its exploration can be useful towards the estimation of mean systemic filling pressure with the circuits time constant, as introduced by Bogatu et al. (2021). As a final remark on this topic, the sensitivity analysis points at cuff length and cuff inflation rate as important determinants of cuff-vasculature interaction, meaning further studies will account for this to observe cuff-induced changes more accurately in PAT/PTT/distal BP oscillations. In addition, parallel studies are seeking to achieve a more direct measurement of the arterial and venous parameters across a broader set of demographics via imaging, while also investigating the mechanisms of pressure transmission via the cuff at brachial site to obtain improved transmural pressure control (Bogatu et al., 2019).

#### 4.2.2 BP value vs. distal response correlation

The results from the simulation indicate a strong linear relationship between distal ∆PTT and PP and SBP. This is an important finding, as PP could indicate the expected change in distal ΔPTT during cuff inflation, therefore allowing for improved estimation of brachial ΔPTT as measured non-invasively via ECG-PPG combination.

When validating this relationship via patient data, we find that, for subjects S1, S2, and S4, an increase in PP is indeed indicative of an increase in *max|∆PTT*_*distal*_ *|*. However, this relationship does not hold for S3, where a decrease in *max|∆PTT*_*distal*_ *|* is found with an increase in PP. This indicates limitations of the current simulation model. While at first sight, the model represents all signal characteristics well, the in-depth sensitivity analysis highlights specifically which aspects require further investigation via dedicated studies.

### 4.3 Limitations of the study and future research

There are several factors which may limit the model-clinical data comparison. For example, motion and breathing artifacts, the low number of beats recorded per inflation and the short length of the cuff all affect the SNR, impacting the assessment of amplitude and onset of the response. Nevertheless, qualitative comparisons between model output and measurements are possible. The cuff-induced vasculature response is observed to follow behaviors which are predicted via the model; the trends in signal changes induced via cuff inflation are accurately represented. A more quantitative, one-on-one model-clinical data assessment is not yet possible also due to the difficulty of obtaining all parameter values specific to one patient. However, alternative measurement modalities such as MRI (Bogatu et al., 2022) may enable such research in the future.

In addition, the current work is focused on response of vasculature to relatively short inflations. A more complete model-clinical data comparison relies on design of new clinical studies. This work contributes to planning of such new investigations and definition of study aims; e.g., the sensitivity analysis reveals how cuff length and duration of inflation are expected to impact vasculature response. It is necessary to conduct focused investigations regarding different cuff inflation strategies (e.g., response to inflation/deflation, inflation speed, cuff length, frequency of occlusions, site at which occlusion is applied). Also, the current study highlights inaccuracies in our understanding of the relationship between systemic BP and distal PTT; future studies can be specifically aimed towards the identified effects.

The model representing distal vasculature (Figure 2) also needs to be studied in the context of multi-segment models which describe the entire circulation (e.g., Avolio, 1980), in order to determine the extent to which reflection coefficients, cardiac output, contractility and general waveform characteristics impact the vasculature response to cuff occlusion.

While the dataset utilized for this work includes a substantial amount of cuff inflation segments (the core “unit” of our study), these were recorded from only 4 subjects, implying limited demographic variability, particularly in terms of cardiovascular health status. It should also be considered that the subjects were undergoing invasive surgical procedures and were anesthetized, two factors that may significantly impact their hemodynamic behavior.

Finally, despite popular and powerful, the variance-based Global Sensitivity Analysis has its weaknesses. An important assumption of the employed method is the independence between the parameters. We tackle this via efforts to create a representative and realistic parameter set. Nevertheless, improved analyses may be performed once a more complete understanding of the potential links between the individual model parameters is acquired.

## 5 Conclusion

Measuring the response of a BP surrogate (PAT/PTT) to transmural pressure modulation controlled via the cuff is a promising approach to improve robustness of calibrations of BP surrogates like PAT and PTT. A detailed understanding of the inflation process and its impact on the hemodynamics distal to the cuff provides insights on how to implement such a calibration strategy.

For that purpose, we investigate a “low complexity” simulation model and compare its performance with real clinical data. Despite its simplicity, the simulation model characteristics agrees qualitatively with experimental findings. The simultaneous onset of distal BP and PTT changes were found to be modelled correctly. However, improved understanding is needed to better model the onset and amplitude of vasculature responses to cuff inflation.

A sensitivity analysis showed that cuff length and cuff inflation rate–both of which can be easily changed in practice - have a significant effect on vasculature response to cuff inflation and may be explored to improve the PAT-BP calibration framework, recommended to be subject of further research. The link between BP and cuff-induced vascular response was also analyzed in depth. A particularly interesting correlation between BP and distal PTT has been revealed, paving the way towards improved BP surrogate calibration. However, a first check with a limited set of patient data gave inconsistent results to be investigated in detail, ideally with an expanded set of patients. While at first sight, the model represents all the signal characteristics well, the in-depth sensitivity analysis highlights specifically which aspects require further investigation via dedicated studies.

Concluding, the model under analysis serves as a valuable tool towards understanding of vascular dynamics occurring during cuff inflation. The model can facilitate the development of monitoring techniques that rely on cuff-based modulation of BP surrogates (PAT/PTT). This study highlighted specifically which effects are/are not well represented via the model, as well as it identified promising avenues via which further investigations can be conducted as well as suggesting improvements of the simulation model.

## Data Availability

The datasets presented in this article are not readily available due to privacy restrictions. Requests to access the datasets should be directed to laura.bogatu@philips.com.
